# Liquid Crystal Ordering in the Hexagonal Phase of Rod-Coil Diblock Copolymers

**DOI:** 10.3390/polym12061262

**Published:** 2020-05-31

**Authors:** Mikhail A. Osipov, Maxim V. Gorkunov, Alexander A. Antonov

**Affiliations:** 1 Department of Mathematics and Statistics, University of Strathclyde, Glasgow G1 1XH, Scotland, UK; 2 Topchiev Institute of Petrochemical Synthesis, Russian Academy of Sciences, 119991 Moscow, Russia; 3 Shubnikov Institute of Crystallography, Federal Scientific Research Centre “Crystallography and Photonics“, Russian Academy of Sciences, 119333 Moscow, Russia; gorkunov@crys.ras.ru (M.V.G.); antonov.wasd@yandex.ru (A.A.A.)

**Keywords:** diblock copolymer, density functional, hexagonal phase

## Abstract

Density functional theory of rod-coil diblock copolymers, developed recently by the authors, has been generalised and used to study the liquid crystal ordering and microphase separation effects in the hexagonal, lamellar and nematic phases. The translational order parameters of rod and coil monomers and the orientational order parameters of rod-like fragments of the copolymer chains have been determined numerically by direct minimization of the free energy. The phase diagram has been derived containing the isotropic, the lamellar and the hexagonal phases which is consistent with typical experimental data. The order parameter profiles as functions of temperature and the copolymer composition have also been determined in different anisotropic phases. Finally, the spatial distributions of the density of rigid rod fragments and of the corresponding orientational order parameter in the hexagonal phase have been calculated.

## 1. Introduction

Rod-coil block copolymers are very interesting soft matter systems which are composed of copolymer macromolecules with flexible and rigid parts of various chemical structure including rod-like fragments of semiconducting polymers [1,2], polypeptides [3,4] and polysaccharides [5,6]. These systems exhibit numerous separated phases such as the orthogonal, tilted and perforated lamellar phases, the hexagonal phase, the gyroid and body-centred cubic structures [7,8,9,10,11,12,13,14,15,16,17]. In these phases, the rod-like fragments are orientationally ordered and thus the rod-coil copolymers combine the properties of coil-coil block copolymers and conventional liquid crystals. The most common phase exhibited by rod-coil copolymers is the lamellar phase which is apparently stabilized both by the microphase separation effects and by the one-dimensional “crystallization” characteristic for smectic liquid crystals. At relatively weak segregation, rod-coil copolymers usually undergo a transition from the isotropic to nematic and then into the orthogonal lamellar phase or directly into the lamellar phase [7,8,9,10] which is similar to liquid crystal materials. At stronger segregation the hexagonal phase may also appear at low temperatures and small fractions of rod segments [7,8]. Rod-coil block copolymers are also very promising materials for applications in polymer photovoltaics [1,18,19] and LEDs [20,21,22].

Formulating a consistent molecular-statistical theory of rod-coil block copolymers is rather challenging as it should account for both translational and orientational degrees of freedom and a large number of the corresponding order parameters. At present, there exist three different approaches to the theory of these systems. The first approach is based on the Landau – de Gennes expansion of the free energy in terms of the translational and the orientational order parameters [23,24]. The coefficients of the free energy expansion have been calculated using the Flory-Huggins theory [23] or the explicit expressions for the monomer-monomer correlation functions of the ideal Gaussian polymer chains [24] which have been derived following the approach proposed by Leibler [25] for coil-coil diblock copolymers. This enables one to describe a number of different phases including the nematic phase, orthogonal lamellar and tilted lamellar phases. On the other hand, the free energy expansion is valid only in the vicinity of the transition into the isotropic phase and in the case of weak segregation: the equilibrium density of monomers contains only one Fourier harmonic and is proportional to the corresponding translational order parameter [23,24]. This theory also does not account for the induced orientational order of rod fragments in the lamellar phase which has been observed experimentally [7,8].

The second approach is based on the generalisation of the self-consistent field theory (SCFT), which has been very successful in the description of both coil-coil block copolymers and rod-coil diblock copolymers [26,27,28,29,30]. In the SCFT, the interaction between different chains is taken into account in the random phase approximation which is similar to the approximation used in Leibler’s theory and its modifications [25,31,32]. The free energy of a single chain in a self-consistent mean-field is calculated by numerically evaluating the path integral along the chain. SCFT is generally computationally challenging, in particular in the case of long polymer chains with orientational degrees of freedom. As a result, in the past the theory has been applied to rod-coil block copolymers using various simplified models including lattice models [26,29], two-dimensional models [28], or the models which do not take into account anisotropic interaction between rods or assume a perfect orientational order [26,27,29]. Recently, however, new computational methods have been developed which enable one to apply the SCFT to 3D systems with orientational degrees of freedom in the general case. For example, Kriksin and Khalatur [30] have applied a large scale parallel 3D SCFT scheme to a rod-coil diblock copolymer with a Gaussian chain and have been able to describe the unusual columnar hexagonal phase with chiral distribution of achiral rods.

There exists also another version of the SCFT which is suitable for rod-coil block copolymers. In this approach, the evaluation of the path integral along the chain is replaced by solving the modified diffusion equations for worm-like chains [33,34,35,36,37,38,39,40,41]. This theory enables one to describe the variety of different phases and to obtain some information about the orientational ordering of rigid fragments. One notes that the chain propagator satisfies a six-dimensional diffusion-like equation and hence this approach also presents significant computational challenges. Nevertheless, recently effective numerical algorithms can be developed (see, for example, [42]) which enable one to describe 3D systems with spatial variation [35,36,37,38,42]. For example, diverse phase diagrams containing lamellar, hexagonal, gyroid and cubic phases have been obtained by Tang et al. [35], the nematic phase as well as the orthogonal and tilted lamellar phases have been described by Song et al. [36] and the effect of rigidity of a semiflexible block on the phase behaviour of semiflexible rod-coil block copolymers has been studied in detail by Li et al. [38]. In this theory, however, the rod-like fragment is formally described as a flexible chain with large persistence length and thus some flexibility of the rods is allowed.

Very recently, the general density functional approach, which is successfully used in the molecular theory of liquid crystals [43,44,45,46,47] and inhomogeneous fluids [48], has been applied by the authors to develop an analytical molecular-statistical theory of rod-coil diblock copolymers [49]. In this approach, the free energy is expressed as a functional of the equilibrium densities of rod and coil monomers which are obtained self-consistently by minimization of the free energy functional. The free energy also depends on the direct correlation functions between rod and coil monomers in the reference system of noninteracting copolymer chains. These correlation functions have been calculated before by various authors in the case of Gaussian chains [25,31,32]. One notes that the theory [49] is based on the self-consistency equations for the translational and orientational order parameters and does not employ the free energy expansion in terms of the order parameters. Thus it may be approximately valid in the case of relatively strong segregation and at low temperatures when the order parameters are not small and the densities are nonlinear functions of the order parameters containing many Fourier harmonics. The theory enables one to calculate numerically the profiles of both translational and orientational order parameters in the orthogonal lamellar phase in a computationally efficient way. At the same time, the theory developed in [49] is restricted to the case of one-dimensional periodicity and thus it cannot be used to describe the hexagonal phase as well as more complicated phases with a three dimensional structure exhibited by rod-coil block copolymers.

In this paper, we propose a more general density functional theory of rod-coil diblock copolymers which enables one to describe the orientational and translational order in the hexagonal phase. The order parameters have been calculated numerically for different cross sections of the phase diagram taking into account the incompressibility condition within the formalism of Lagrange multipliers.

The paper is arranged as follows. In Section 2, the density functional approach to the molecular theory of rod-coil diblock copolymers is presented. The explicit expressions for the free energy of the lamellar and hexagonal phases in terms of the corresponding order parameters are presented in Section 3 and Section 4, respectively, together with the corresponding expressions for the equilibrium number densities of rod and coil monomers. In Section 5, the numerical minimization of the free energy is discussed and the results are presented including the profiles of orientational and translation order parameters in the hexagonal phase and the spatial distribution of the density and the orientational order of rods. Section 6 contains our conclusions.

## 2. Density Functional Approach to the Theory of Block Copolymers

Consider a rod-coil diblock copolymer consisting of *M* identical molecules composed of *N* monomers. Each molecular chain includes two parts: one containing $Nf_{c}$ coil monomers and the other containing $Nf_{r}=N\left(1-f_{c}\right)$ rod monomers. A general molecular-statistical theory of such polymers can be derived in a consistent way using the density functional approach which has been widely used in the theory of liquid crystals [43,44,45,46,47,50]. In this approach, the free energy *F* of the diblock copolymer is a functional of the one-particle densities, $\rho_{c}\left(r\right)$ and $\rho_{r}\left(r,a\right)$, which are defined as thermal averages of the microscopic densities $\rho_{c}^{M}\left(r\right)$ and $\rho_{r}^{M}\left(r,a\right)$ of the coils and rods correspondingly. The unit vector a here stands for the direction of the long axis of a rod fragment and the indices *r* and *c* denote rods and coils, respectively.

The general structure of the free energy functional F[ρ(r,a)] is unknown, but its functional derivatives are related to the direct correlation functions of the system. Then the free energy of the anisotropic phase can be obtained by performing a functional Taylor expansion of the free energy around its value in the isotropic phase. As discussed in detail in [49] the free energy functional of the rod-coil diblock copolymers can approximately be expressed as:

(1) $$\begin{array}{c} \beta F=\beta F_{I}+\int \rho_{c}\left(r\right)\left[\ln\rho_{c}\left(r\right)-1\right]dr+\int \rho_{r}\left(r,a\right)\left[\ln\rho_{r}\left(r,a\right)-1\right]drda\\ +\int \chi \left(r_{12}\right)\delta \rho_{r}\left(r_{1},a_{1}\right)\delta \rho_{c}\left(r_{2}\right)dr_{1}dr_{2}da_{1}-\frac{1}{2}\beta \int J\left(r_{12}\right)P_{2}\left(a_{1}\cdot a_{2}\right)\delta \rho_{r}\left(r_{1},a_{1}\right)\delta \rho_{r}\left(r_{2},a_{2}\right)dr_{1}dr_{2}da_{1}da_{2}\\ -\frac{1}{2}\sum_{\nu ,\eta =r,c}\int C_{\nu ,\eta }\left(r_{12},a_{1},a_{2}\right)\delta \rho_{\nu }\left(r_{1},a_{1}\right)\delta \rho_{\eta }\left(r_{2},a_{2}\right)dr_{1}dr_{2}da_{1}da_{2} \end{array}$$

where $F_{I}$ is the free energy of the isotropic phase, $C_{cc}\left(r_{12}\right)$, $C_{rc}\left(r_{12},a_{1}\right)$ and $C_{rr}\left(r_{12},a_{1},a_{2}\right)$ are the coil-coil, rod-coil and rod-rod direct correlation functions calculated in the reference disordered phase of noninteracting copolymer chains, and $\delta \rho =\rho -\rho_{0}$ is the difference between the one-particle densities in the inhomogeneous phase and the density of the isotropic phase. Here the interchain interactions are taken into account in the molecular field approximation, including the repulsion between rod and coil monomers, specified by the Flory-Huggins parameter χ, and the Maier-Saupe orientational interaction potential between the rods $J\left(r_{12}\right)P_{2}\left(a_{1}\cdot a_{2}\right)$.

Minimization of the free energy (1) with respect to the number densities of rod and coil monomers yields the following self-consistent equations for $\rho_{c}\left(r_{1}\right)$ and $\rho_{r}\left(r_{1},a\right)$:

(2) $$\rho_{c}\left(r_{1}\right)=Z_{c}^{-1}\exp\left\{\int C_{cc}\left(r_{12}\right)\delta \rho_{c}\left(r_{2}\right)dr_{2}\right.-\left.\int \left[\chi \left(r_{12}\right)-C_{rc}\left(r_{12},a\right)\right]\delta \rho_{r}\left(r_{2},a\right)dr_{2}da\right\}$$

(3) $$\begin{array}{c} \rho_{r}\left(r_{1},a_{1}\right)=Z_{r}^{-1}\exp\left\{\int C_{rr}\left(r_{12},a_{1},a_{2}\right)\delta \rho_{r}\left(r_{2},a\right)dr_{2}da_{2}-\int \left[\chi \left(r_{12}\right)-C_{rc}\left(r_{12},a_{1}\right)\right]\delta \rho_{c}\left(r_{2}\right)dr_{2}\right.\\ +\left.\beta \int J\left(r_{12}\right)P_{2}\left(a_{1}\cdot a_{2}\right)\delta \rho_{r}\left(r_{2},a_{2}\right)dr_{2}da_{2}\right\} \end{array}$$

where $Z_{c}$ and $Z_{r}$ are the corresponding normalization factors.

One notes that in the density functional approach the equilibrium densities of rod and coil monomers are determined by the self-consistent nonlinear integral Equations (2) and (3), and thus they are not described by a simple cosine-like profile as in the Leibler theory. In the general case, these equations contain many Fourier harmonics and the whole set of wave vectors. A similar self-consistent equation is also used to derive the well known Maier-Saupe model for low molecular weight nematic liquid crystals [50], in which the one-particle density is the phase space is proportional to the orientational distribution function.

In Equations (1)–(3) the direct pair correlation functions $C_{\nu ,\eta }\;\left(\nu ,\eta =r,c\right)$ of the reference disordered phase of noninteracting chains are related to the corresponding full pair correlation function $h_{\nu ,\eta }\left(r_{12},a\right)$ by the Ornstein-Zernike equation [49]:

(4) $$h_{\nu ,\eta }\left(r_{12},a\right)=C_{\nu ,\eta }\left(r_{12},a\right)+\rho_{\eta }\int C_{\nu ,\gamma }\left(r_{13},a\right)h_{\eta ,\gamma }\left(r_{23},a\right)dr_{3},$$

and $\rho_{\nu }$ is the average number density of the component ν. One notes that the correlation functions in Equation (4) are the intrachain correlation functions and thus they depend on the unit vector a along the rigid part of the corresponding macromolecule. In the reference system of noninteracting chains there are no correlations between rod monomers in different copolymer chains with different orientations $a_{1}$ and $a_{2}$.

The full pair correlation function $h_{\nu ,\eta }\left(r_{12},a\right)$ in Equation (4) is related to the two-particle distribution function $f_{\nu ,\eta }\left(r_{12},a\right)$ as

(5) $$f_{\nu ,\eta }\left(r_{12},a\right)=f_{\nu }\left(r_{12},a\right)f_{\eta }\left(r_{12},a\right)\left[h_{\nu ,\eta }\left(r_{12},a\right)+1\right],$$

where $f_{\nu ,\eta }\left(r_{12},a\right)$ can be defined as [51]:

(6) $$\rho_{\nu }\rho_{\eta }f_{\nu ,\eta }\left(r_{12},a\right)=\langle \sum_{i\neq j=1}^{N}\delta \left(x_{1}-x_{i}^{\nu }\right)\delta \left(x_{2}-x_{j}^{\eta }\right)\rangle ,$$

where x=(r,a).

Finally it is possible to establish a relationship [49] between the two-particle distribution functions $f_{\nu ,\eta }\left(r_{12},a\right)$ and the density-density correlation functions $G_{\nu ,\eta }\left(r_{12},a\right)$ which are defined as [25]:

(7) $$G_{\nu ,\gamma }\left(r_{12},a\right)=\langle \delta \rho_{\nu }^{M}\left(x_{1}\right)\delta \rho_{\gamma }^{M}\left(x_{2}\right)\rangle ,$$

where $\delta \rho_{\nu }^{M}\left(x\right)=\rho_{\nu }^{M}\left(x\right)-\rho_{\nu }$. and $\rho_{\nu }^{M}\left(x\right)$ is the microscopic number density of monomers ν which is expressed as:

(8) $$\rho_{\nu }^{M}\left(x\right)=\sum_{i}\delta \left(x-x_{i}\right).$$

Substituting Equation (8) into Equation (7) one obtains

(9) $$\begin{array}{c} G_{\nu ,\eta }\left(r_{12},a\right)=\langle \rho_{\nu }^{M}\left(r_{1},a\right)\rho_{\eta }^{M}\left(r_{2},a\right)\rangle -\rho_{\nu }\rho_{\eta }\\ =\langle \sum_{i\neq j=1}^{N}\delta \left(x_{1}-x_{i}^{\nu }\right)\delta \left(x_{2}-x_{j}^{\eta }\right)\rangle +\delta_{\nu ,\eta }\langle \sum_{i=1}^{N}\delta \left(x_{1}-x_{i}^{\nu }\right)\delta \left(x_{2}-x_{i}^{\nu }\right)\rangle -\rho_{\nu }\rho_{\eta }\\ =\rho_{\nu }\rho_{\eta }f_{\nu ,\eta }\left(r_{12},a\right)-\rho_{\nu }\rho_{\eta }+\rho_{\nu }\delta_{\nu ,\eta }\delta \left(x_{2}-x_{1}\right)=\rho_{\nu }\rho_{\eta }h_{\nu ,\eta }\left(r_{12},a\right)+\rho_{\nu }\delta_{\nu ,\eta }\delta \left(x_{2}-x_{1}\right), \end{array}$$

In the Fourier representation, the Ornstein-Zernike Equation (4) can be written in the following form:

(10) $$h_{\nu ,\eta }\left(q,a\right)=C_{\nu ,\eta }\left(q,a\right)+\rho_{\gamma }h_{\nu ,\gamma }\left(q,a\right)C_{\gamma ,\eta }\left(q,a\right).$$

Equation (10) can be solved in the matrix form which enables one to obtain the following expression for the direct correlation functions:

(11) $$C_{\nu ,\gamma }\left(q,a\right)=h_{\nu ,\eta }\left(q,a\right){\left[\delta_{\gamma ,\eta }+\rho_{\gamma }h_{\gamma ,\eta }\left(q,a\right)\right]}^{-1}=\rho_{\nu }^{-1}\delta_{\nu ,\gamma }-{\left[\rho_{\nu }\delta_{\nu ,\gamma }+\rho_{\nu }\rho_{\gamma }h_{\nu ,\gamma }\left(q,a\right)\right]}^{-1}.$$

The density-density correlation function between the monomers in the reference disordered phase can also be expressed in the Fourier representation:

(12) $$G_{\nu ,\gamma }\left(q,a\right)=\rho_{\nu }\delta_{\nu ,\gamma }+\rho_{\nu }\rho_{\gamma }h_{\nu ,\gamma }\left(q,a\right).$$

Combining Equations (12) and (11) one obtains the expression for the direct correlation functions $C_{\nu ,\gamma }\left(q,a\right)$ in terms of the density-density correlation functions $G_{\nu ,\gamma }\left(r_{12},a\right)$:

(13) $$C_{\nu ,\gamma }\left(q,a\right)=\rho_{\nu }^{-1}\delta_{\nu ,\gamma }-G_{\nu ,\gamma }{\left(q,a\right)}^{-1}.$$

The density correlation functions $G_{\nu ,\gamma }\left(q,a\right)$ for the system of non-interacting Gaussian rod-coil copolymer chains have been calculated before [24,49] and are presented in Appendix A.

Equations (2) and (3) can be specified further using some approximations [49] which lead to different expressions in the lamellar and the hexagonal phases. The corresponding approximate expressions for the free energy of these phase are derived below.

## 3. Free Energy of the Orthogonal Lamellar Phase

In the orthogonal lamellar phase, the one-particle densities should be periodic functions with the period of the phase. Hence all “mean-field” potentials, i.e., all terms in the exponential functions in Equations (2) and (3) are also periodic with the same period and can be expanded in the Fourier series. Thus in the first approximation the corresponding terms can be approximated by the following first terms of the Fourier expansion:

(14) $$\int C_{cc}\left(r_{12}\right)\delta \rho_{c}\left(r_{2}\right)dr_{2}\approx \rho_{0}f_{c}\psi_{c}C_{cc}\left(q\right)\cos\left(q\cdot r_{1}\right)$$

(15) $$\int C_{rr}\left(r_{12},a_{1},a_{2}\right)\delta \rho_{r}\left(r_{2},a_{2}\right)dr_{2}da_{2}\approx \rho_{0}f_{r}\cos\left(q\cdot r_{1}\right)\int C_{rr}\left(q,a_{1},a_{2}\right)\cos\left(q\cdot r_{2}\right)\delta \rho_{r}\left(r_{2},a_{2}\right)dr_{2}da_{2}$$

(16) $$\int C_{rc}\left(r_{12},a_{1}\right)\delta \rho_{c}\left(r_{2}\right)dr_{2}\approx \rho_{0}f_{c}\psi_{c}C_{rc}\left(q,a_{1}\right)\cos\left(q\cdot r_{1}\right)$$

(17) $$\int C_{rc}\left(r_{12},a_{2}\right)\delta \rho_{r}\left(r_{2},a_{2}\right)dr_{2}da_{2}\approx \rho_{0}f_{r}\cos\left(q\cdot r_{1}\right)\int C_{rc}\left(q,a_{2}\right)\cos\left(q\cdot r_{2}\right)\delta \rho_{r}\left(r_{2},a_{2}\right)dr_{2}da_{2},$$

and

(18) $$\int J\left(r_{12}\right)P_{2}\left(a\cdot a_{2}\right)\delta \rho_{r}\left(r_{2},a_{2}\right)dr_{2}da_{2}\approx \rho_{0}f_{r}\left\{J_{0}SP_{2}\left(a\cdot k\right)\right.\left.+J_{2}\sigma P_{2}\left(a\cdot k\right)\cos\left(q\cdot r_{1}\right)\right\}.$$

Here k is the unit vector along q and the order parameters are defined by the following expressions:

(19) $$\psi_{c}={\langle \cos(q\cdot r)\rangle }_{c}=\int f_{1}^{\left(c\right)}\left(r\right)\cos\left(q\cdot r\right)dr,$$

(20) $$\sigma ={\langle P_{2}\left(a\cdot k\right)\cos\left(q\cdot r\right)\rangle }_{r}=\int f_{1}^{\left(r\right)}\left(r,a\right)P_{2}\left(a\cdot k\right)\cos\left(q\cdot r\right)drda,$$

(21) $$S={\langle P_{2}\left(a\cdot k\right)\rangle }_{r}=\int f_{1}^{\left(r\right)}\left(r,a\right)P_{2}\left(a\cdot k\right)drda$$

and the averaging is performed with the one-particle distribution functions $f_{1}^{\left(c\right)}\left(r\right)$ and $f_{1}^{\left(r\right)}\left(r,a\right)$ ($\rho_{\nu }=Mf_{\nu }f_{1}^{\left(\nu \right)}$) of rod and coil fragments respectively.

One notes that rod-rod and rod-coil density correlation functions depend on the orientation of the unit vector a along the rod segment with respect to the wave vector q. This orientational dependence is rather complicated and in the first approximation it is possible to expand the correlation function in Legendre polynomials $P_{2n}\left(k\cdot a\right)$ keeping the first few terms:

(22) $$C_{cc}\left(q\right)\approx C_{cc}^{\left(0\right)}\left(q\right)$$

(23) $$C_{rc}\left(q,a\right)\approx C_{rc}^{\left(0\right)}\left(q\right)+C_{rc}^{\left(2\right)}\left(q\right)P_{2}\left(a\cdot k\right)$$

(24) $$C_{rr}\left(q,a_{1},a_{2}\right)\approx C_{rr}^{\left(0\right)}\left(q\right)+\frac{1}{2}C_{rr}^{\left(2\right)}\left(q\right)\left[P_{2}\left(a_{1}\cdot k\right)+P_{2}\left(a_{2}\cdot k\right)\right],$$

where q=|q|, $C_{\nu \eta }^{\left(0\right)}\left(q\right)=\int C_{\nu \eta }\left(q,a\right)da$ and $C_{\nu \eta }^{\left(2\right)}\left(q\right)$ is determined from the expression: $C_{\nu \eta }\left(q,a=k\right)=C_{\nu \eta }^{\left(0\right)}\left(q\right)+C_{\nu \eta }^{\left(2\right)}\left(q\right)$. Here we have taken into account that the rod-rod correlation function must be symmetric.

Substituting Equations (22)–(24) into Equations (15)–(17) one obtains the following explicit expressions for the corresponding integrals in terms of the order parameters:

(25) $$\begin{array}{c} \int C_{rr}\left(r_{12},a,a_{2}\right)\delta \rho_{r}\left(r_{2},a_{2}\right)dr_{2}da_{2}\\ \approx \rho_{0}f_{r}\psi_{r}\left[C_{rr}^{\left(0\right)}\left(q\right)\cos\left(q\cdot r_{1}\right)+\frac{1}{2}C_{rr}^{\left(2\right)}\left(q\right)P_{2}\left(a\cdot k\right)\cos\left(q\cdot r_{1}\right)\right]+\frac{1}{2}\rho_{0}f_{r}\sigma C_{rr}^{\left(2\right)}\left(q\right)\cos\left(q\cdot r_{1}\right) \end{array}$$

(26) $$\int C_{rc}\left(r_{12},a\right)\delta \rho_{c}\left(r_{2}\right)dr_{2}\approx \rho_{0}f_{c}\psi_{c}\left[C_{rc}^{\left(0\right)}\left(q\right)\cos\left(q\cdot r_{1}\right)\right.\left.+C_{rc}^{\left(2\right)}\left(q\right)P_{2}\left(a\cdot k\right)\cos\left(q\cdot r_{1}\right)\right]$$

(27) $$\int C_{rc}\left(r_{12},a\right)\delta \rho_{r}\left(r_{2},a\right)dr_{2}da\approx \rho_{0}f_{r}\cos\left(q\cdot r_{1}\right)\left[\psi_{r}C_{rc}^{\left(0\right)}\left(q\right)+\sigma C_{rc}^{\left(2\right)}\left(q\right)\right],$$

where we have set $a_{1}=a_{2}=a$ in the final expressions because all rod segments are always parallel within one macromolecule. Also a positional order parameter of rods is introduced as:

(28) $$\psi_{r}={\langle \cos(q\cdot r)\rangle }_{r}=\int f_{1}^{\left(r\right)}\left(r,a\right)\cos\left(q\cdot r\right)drda.$$

Finally, the equilibrium densities of rod and coil monomers in the orthogonal lamellar phase can be explicitly expressed in terms of the translational and orientational order parameters (19)–(21) and (28) using Equations (14) and (25)–(27):

(29) $$\rho_{c}\left(r\right)=Z_{c}^{-1}\exp\left\{\rho_{0}\cos\left(q\cdot r\right)\left[f_{c}C_{cc}^{\left(0\right)}\left(q\right)\psi_{c}+f_{r}C_{rc}^{\left(0\right)}\left(q\right)\psi_{r}+f_{r}C_{rc}^{\left(2\right)}\left(q\right)\sigma -f_{r}\chi \psi_{r}\right]\right\}$$

(30) $$\begin{array}{c} \rho_{r}\left(r,a\right)=Z_{r}^{-1}\exp\left\{\rho_{0}\cos\left(q\cdot r\right)\left[f_{r}C_{rr}^{\left(0\right)}\left(q\right)\psi_{r}+f_{c}C_{rc}^{\left(0\right)}\left(q\right)\psi_{c}-f_{c}\chi \psi_{c}\right]\right.\\ +\rho_{0}P_{2}\left(a\cdot k\right)\cos\left(q\cdot r\right)\left[f_{c}C_{rc}^{\left(2\right)}\left(q\right)\psi_{c}+\frac{1}{2}f_{r}C_{rr}^{\left(2\right)}\left(q\right)\psi_{r}+f_{r}\beta J_{2}\sigma \right]\\ +\left.\frac{1}{2}\rho_{0}C_{rr}^{\left(2\right)}\left(q\right)f_{r}\sigma \cos\left(q\cdot r\right)+\rho_{0}f_{r}\beta J_{0}SP_{2}\left(a\cdot k\right)\right\} \end{array}$$

Substituting Equations (29) and (30) back into the free energy functional (1) one obtains the following explicit expression for the free energy of the lamellar phase:

(31) $$\begin{array}{c} \beta F/V=\frac{1}{2}\rho_{0}^{2}\sigma \left[f_{r}^{2}C_{rr}^{\left(2\right)}\left(q\right)\psi_{r}+2f_{c}f_{r}C_{rc}^{\left(2\right)}\left(q\right)\psi_{c}\right]+\frac{1}{2}\beta \rho_{0}^{2}S^{2}f_{r}^{2}J_{0}+\frac{1}{2}\beta \rho_{0}^{2}f_{r}^{2}J_{2}\sigma^{2}\\ +\frac{1}{2}\rho_{0}^{2}f_{r}^{2}C_{rr}^{\left(0\right)}\left(q\right)\psi_{r}^{2}+\frac{1}{2}\rho_{0}^{2}f_{c}^{2}C_{cc}^{\left(0\right)}\left(q\right)\psi_{c}^{2}+\rho_{0}^{2}f_{r}f_{c}C_{rc}^{\left(0\right)}\left(q\right)\psi_{r}\psi_{c}-\rho_{0}^{2}f_{r}f_{c}\chi \psi_{c}\psi_{r}-\rho_{0}f_{r}\ln Z_{r}-\rho_{0}f_{c}\ln Z_{c} \end{array}$$

where *V* is the polymer volume and

(32) $$Z_{c}=\int dz\exp\left[\rho_{0}\cos\left(qz\right)\left(f_{c}C_{cc}^{\left(0\right)}\left(q\right)\psi_{c}\right.\right.\left.\left.+\;f_{r}C_{rc}^{\left(2\right)}\left(q\right)\sigma +f_{r}C_{rc}^{\left(0\right)}\left(q\right)\psi_{r}-f_{r}\chi \psi_{r}\right)\right],$$

(33) $$\begin{array}{c} Z_{r}=\int dzda\exp\left[\rho_{0}\cos\left(qz\right)\left(f_{r}C_{rr}^{\left(0\right)}\left(q\right)\psi_{r}+f_{c}C_{rc}^{\left(0\right)}\left(q\right)\psi_{c}-f_{c}\chi \psi_{c}\right)\right.\\ +\rho_{0}\cos\left(qz\right)P_{2}\left(a\cdot k\right)\left(f_{c}C_{rc}^{\left(2\right)}\left(q\right)\psi_{c}+\frac{1}{2}f_{r}C_{rr}^{\left(2\right)}\left(q\right)\psi_{r}+f_{r}\beta J_{2}\sigma \right)\\ \left.+\frac{1}{2}\cos\left(qz\right)\rho_{0}f_{r}C_{rr}^{\left(2\right)}\left(q\right)\sigma +\rho_{0}f_{r}\beta J_{0}SP_{2}\left(a\cdot k\right)\right], \end{array}$$

where $J_{0}>0,\chi >0$ and the *z*-axis is oriented along the vector q. The direct correlation functions $C^{\left(0\right)}\left(q\right)$ and $C^{\left(2\right)}\left(q\right)$ are considered in the following section.

## 4. Free Energy of the Hexagonal Phase

The $C_{6h}$ symmetry of the hexagonal phase is determined by a combination of three equivalent wave vectors $q_{1},q_{2},q_{3}$ where $q_{1}+q_{2}+q_{3}=0$ and $|q_{1}\left|=\right|q_{2}\left|=\right|q_{3}|=q$ and hence the one-particle densities of rod and coil monomers should be periodic functions along the corresponding three equivalent directions. Thus every term in the exponential functions in the general Equations (2) and (3) is also periodic and can be expanded in the corresponding Fourier series keeping for simplicity the first terms of the Fourier expansion along these three directions. Similarly to the lamellar phase, the direct correlation functions in Equations (2) and (3) can also be expanded in Legendre polynomials $P_{2n}\left(k_{i}\cdot a\right)$ keeping the first nontrivial terms (see Equations (14)–(17)) for all three $k_{i}$, where $k_{i}=q_{i}/q,i=1,2,3$. As a result one obtains the following approximate expressions for the corresponding terms in the mean-field potentials:

(34) $$\int \chi \left(r_{12}\right)\delta \rho_{\nu }\left(r_{2},a\right)dr_{2}da\approx \rho_{0}f_{\nu }\chi \psi_{\nu }\sum_{i=1}^{3}\cos\left(q_{i}\cdot r_{1}\right)$$

(35) $$\int C_{cc}\left(r_{12}\right)\delta \rho_{c}\left(r_{2}\right)dr_{2}\approx \rho_{0}f_{c}\psi_{c}C_{cc}^{\left(0\right)}\left(q\right)\sum_{i=1}^{3}\cos\left(q_{i}\cdot r_{1}\right)$$

(36) $$\begin{array}{c} \int C_{rr}\left(r_{12},a,a_{2}\right)\delta \rho_{r}\left(r_{2},a_{2}\right)dr_{2}da_{2}\\ \approx \rho_{0}f_{r}\psi_{r}\left[C_{rr}^{\left(0\right)}\left(q\right)\sum_{i=1}^{3}\cos\left(q_{i}\cdot r_{1}\right)+\frac{1}{2}C_{rr}^{\left(2\right)}\left(q\right)\sum_{i=1}^{3}P_{2}\left(a\cdot k_{i}\right)\cos\left(q_{i}\cdot r_{1}\right)\right]\\ +\frac{1}{2}\rho_{0}f_{r}C_{rr}^{\left(2\right)}\left(q\right)\sigma_{k}\sum_{i=1}^{3}\cos\left(q_{i}\cdot r_{1}\right) \end{array}$$

(37) $$\int C_{rc}\left(r_{12},a\right)\delta \rho_{c}\left(r_{2}\right)dr_{2}\approx \rho_{0}f_{c}\psi_{c}\left[C_{rc}^{\left(0\right)}\left(q\right)\sum_{i=1}^{3}\cos\left(q_{i}\cdot r_{1}\right)\right.\left.+C_{rc}^{\left(2\right)}\left(q\right)\sum_{i=1}^{3}P_{2}\left(a\cdot k_{i}\right)\cos\left(q_{i}\cdot r_{1}\right)\right]$$

(38) $$\int C_{rc}\left(r_{12},a\right)\delta \rho_{r}\left(r_{2},a\right)dr_{2}da\approx \rho_{0}f_{r}\sum_{i=1}^{3}\cos\left(q_{i}\cdot r_{1}\right)\times \left[\psi_{r}C_{rc}^{\left(0\right)}\left(q\right)+\sigma_{k}C_{rc}^{\left(2\right)}\left(q\right)\right],$$

(39) $$\int J\left(r_{12}\right)P_{2}\left(a\cdot a_{2}\right)\delta \rho_{r}\left(r_{2},a_{2}\right)dr_{2}da_{2}\approx \rho_{0}f_{r}J_{0}SP_{2}\left(a\cdot n\right)+\rho_{0}f_{r}J_{2}\sigma P_{2}\left(a\cdot n\right)\sum_{i=1}^{3}\cos\left(q_{i}\cdot r_{1}\right),$$

where n is the macroscopic director in the hexagonal phase and the translational-orientational order parameters $\sigma_{k}$ and σ are expressed as

(40) $$\sigma_{k}={\langle P_{2}\left(a\cdot k_{i}\right)\cos\left(q_{i}\cdot r\right)\rangle }_{r},$$

(41) $$\sigma ={\langle P_{2}\left(a\cdot n\right)\cos\left(q_{i}\cdot r\right)\rangle }_{r}.$$

The orientational order parameter *S* is given by the standard expression:

(42) $$S={\langle P_{2}\left(a\cdot n\right)\rangle }_{r}.$$

According to the $C_{6h}$ symmetry of the hexagonal phase any macroscopic second rank tensor, averaged over the whole system, must be uniaxial. Hence the director n, which specifies the predominant orientation of the rods, must be parallel to axis of the cylinders. Another possibility corresponds to an inhomogeneous isotropic distribution of the director in the plane perpendicular to the macroscopic axis of the hexagonal phase. In the latter case the rods are predominantly perpendicular to the axis of the cylinders. One notes that in both cases $\sigma_{k}\neq \sigma$ and both $\sigma_{k}$ and σ are independent of *i* as all vectors $q_{i}$ are equivalent. In this paper we focus on the more realistic case when n is parallel to the symmetry axis.

Substituting Equations (34)–(39) into the general Equations (2) and (3) one obtains the expressions for the equilibrium number densities of rod and coil monomers in the hexagonal phase:

(43) $$\rho_{c}\left(r\right)=Z_{c}^{-1}\exp\left\{\rho_{0}\sum_{i=1}^{3}\cos\left(q_{i}\cdot r\right)\left[f_{c}C_{cc}^{\left(0\right)}\left(q\right)\psi_{c}+f_{r}C_{rc}^{\left(0\right)}\left(q\right)\psi_{r}+f_{r}C_{rc}^{\left(2\right)}\left(q\right)\sigma_{k}-f_{r}\chi \psi_{r}\right]\right\}$$

(44) $$\begin{array}{c} \rho_{r}\left(r,a\right)=Z_{r}^{-1}\exp\left\{\rho_{0}\sum_{i=1}^{3}\cos\left(q_{i}\cdot r\right)\right.\left[f_{r}C_{rr}^{\left(0\right)}\left(q\right)\psi_{r}+f_{c}C_{rc}^{\left(0\right)}\left(q\right)\psi_{c}-f_{c}\chi \psi_{c}\right]\\ +\rho_{0}\sum_{i=1}^{3}P_{2}\left(a\cdot k_{i}\right)\cos\left(q_{i}\cdot r\right)\left[f_{c}C_{rc}^{\left(2\right)}\left(q\right)\psi_{c}+\frac{1}{2}f_{r}C_{rr}^{\left(2\right)}\left(q\right)\psi_{r}\right]\\ \left.+\rho_{0}f_{r}\beta J_{2}\sigma P_{2}\left(a\cdot n\right)\sum_{i=1}^{3}\cos\left(q_{i}\cdot r\right)+\frac{1}{2}\rho_{0}C_{rr}^{\left(2\right)}\left(q\right)f_{r}\sigma_{k}\sum_{i=1}^{3}\cos\left(q_{i}\cdot r\right)+\rho_{0}f_{r}\beta J_{0}SP_{2}\left(a\cdot n\right)\right\}. \end{array}$$

Substitution of Equations (43) and (44) into the general Equation (1) yields the final expression for the free energy of the hexagonal phase:

(45) $$\begin{array}{c} \frac{\beta F}{V}=\frac{3}{2}\rho_{0}^{2}\left[C_{cc}^{\left(0\right)}\left(q\right)\psi_{c}^{2}f_{c}^{2}+2C_{rc}^{\left(0\right)}\left(q\right)\psi_{c}\psi_{r}f_{c}f_{r}+C_{rr}^{\left(0\right)}\left(q\right)\psi_{r}^{2}f_{r}^{2}\right.\\ \left.+2C_{rc}^{\left(2\right)}\left(q\right)\psi_{c}\sigma_{k}f_{c}f_{r}+C_{rr}^{\left(2\right)}\left(q\right)\psi_{r}\sigma_{k}f_{r}^{2}-2\chi \psi_{c}\psi_{r}f_{c}f_{r}+\beta J_{2}\sigma^{2}f_{r}^{2}\right]\\ +\frac{1}{2}\rho_{0}^{2}f_{r}^{2}\beta J_{0}S^{2}-\rho_{0}\left(f_{c}\ln Z_{c}+f_{r}\ln Z_{r}\right), \end{array}$$

where

(46) $$Z_{c}=\int dr\exp\left\{\rho_{0}\sum_{i=1}^{3}\cos\left(q_{i}\cdot r\right)\left[f_{c}C_{cc}^{\left(0\right)}\left(q\right)\psi_{c}+f_{r}C_{rc}^{\left(0\right)}\left(q\right)\psi_{r}+f_{r}C_{rc}^{\left(2\right)}\left(q\right)\sigma_{k}-f_{r}\chi \psi_{r}\right]\right\}$$

and

(47) $$\begin{array}{c} Z_{r}=\int drda\exp\left\{\rho_{0}\sum_{i=1}^{3}\cos\left(q_{i}\cdot r\right)\right.\left[f_{r}C_{rr}^{\left(0\right)}\left(q\right)\psi_{r}+f_{c}C_{rc}^{\left(0\right)}\left(q\right)\psi_{c}-f_{c}\chi \psi_{c}\right]\\ +\rho_{0}\sum_{i=1}^{3}P_{2}\left(a\cdot k_{i}\right)\cos\left(q_{i}\cdot r\right)\left[f_{c}C_{rc}^{\left(2\right)}\left(q\right)\psi_{c}+\frac{1}{2}f_{r}C_{rr}^{\left(2\right)}\left(q\right)\psi_{r}\right]\\ +\left.\rho_{0}f_{r}\beta J_{2}\sigma P_{2}\left(a\cdot n\right)\sum_{i=1}^{3}\cos\left(q_{i}\cdot r\right)+\frac{1}{2}\rho_{0}C_{rr}^{\left(2\right)}\left(q\right)f_{r}\sigma_{k}\sum_{i=1}^{3}\cos\left(q_{i}\cdot r\right)+\rho_{0}f_{r}\beta J_{0}SP_{2}\left(a\cdot n\right)\right\}. \end{array}$$

To further simplify the free energy, we make an assumption about a relationship between the order parameters σ and $\sigma_{k}$ which is to be verified later. We assume that the orientational distribution function of rods in the hexagonal phase is uniaxial and hence it depends only on the angle θ between the unit vector a in the direction of the rod axis and the director n. One notes that the order parameter σ depends only on the angle θ while the order parameter $\sigma_{k}$ depends also on the azimuthal angle ϕ which specifies the rotation of the unit vector a about the director n:

(48) $$\sigma ={\langle P_{2}\left(\cos\theta \right)\cos\left(q_{i}\cdot r\right)\rangle }_{r},$$

(49) $$\sigma_{k}={\langle \left(\frac{3}{2}\sin^{2}\theta \cos^{2}\phi -\frac{1}{2}\right)\cos\left(q_{i}\cdot r\right)\rangle }_{r}.$$

For the distribution function independent of ϕ, one obtains

(50) $$\sigma_{k}={\langle \left(\frac{3}{4}\sin^{2}\theta -\frac{1}{2}\right)\cos\left(q_{i}\cdot r\right)\rangle }_{r}=-\frac{1}{2}{\langle P_{2}\left(\cos\theta \right)\cos\left(q_{i}\cdot r\right)\rangle }_{r}=-\frac{1}{2}\sigma .$$

This approximate result enables one to substitute the relationship $\sigma \approx -2\sigma_{k}$ into the free energy (45) which can subsequently be minimized with respect to just four order parameters.

## 5. Minimization of the Free Energy and Phase Diagrams

The values of the orientational and translation order parameters at a given temperature in a particular phase can be obtained by minimization of the corresponding free energy given by Equation (31) for the lamellar phase and by Equation (45) for the hexagonal phase. The values of the order parameters are then used to evaluate the free energy of each phase and to derive the phase diagram. Such a minimization has been undertaken using the incomressibility constraint $f_{c}\psi_{c}=-f_{r}\psi_{r}$. This constraint is consistently taken into account using the method of Lagrange multipliers. The constrained free energy of the rod-coil diblock copolymer then contains the additional contribution which depends on the Lagrange multiplier λ:

(51) $$\beta F=\beta F_{0}\left(\rho_{c}\left(r\right),\rho_{r}\left(r,a\right)\right)+\lambda \left[\int \cos\left(q\cdot r\right)\rho_{r}\left(r,a\right)drda\right.\left.+\int \cos\left(q\cdot r\right)\rho_{c}\left(r\right)dr\right],$$

where $\beta F_{0}\left(\rho_{c}\left(r\right),\rho_{r}\left(r,a\right)\right)$ is given by the general expression (1). Here we have taken into account that $N_{\nu }^{-1}\rho_{\nu }\left(r,a\right)=f_{\nu }\left(r,a\right)$, where $f_{\nu }\left(r,a\right)$ is the corresponding one-particle distribution function.

Minimization of the free energy functional (51) yields the equilibrium number densities of rod and coil monomers as functions of the parameter λ:

(52) $$\rho_{c}^{\left(\lambda \right)}\left(r\right)=\frac{1}{Z_{c}^{\left(\lambda \right)}}\exp\left[U_{MF}^{\left(c\right)}\left(r\right)-\lambda \cos\left(q\cdot r\right)\right]$$

(53) $$\rho_{r}^{\left(\lambda \right)}\left(r,a\right)=\frac{1}{Z_{r}^{\left(\lambda \right)}}\exp\left[U_{MF}^{\left(r\right)}\left(r,a\right)-\lambda \cos\left(q\cdot r\right)\right],$$

where $U_{MF}^{\left(c\right)}\left(r\right)$ and $U_{MF}^{\left(r\right)}\left(r,a\right)$ are the mean-field potentials acting on the rod and coil monomers, respectively (i.e., the expressions in the exponential functions in Equations (29) and (30) or (43) and (44). Parameter λ satisfies the following integral equation which is solved numerically:

(54) $$\int \cos\left(q\cdot r\right)\rho_{r}^{\left(\lambda \right)}\left(r,a\right)drda=-\int \cos\left(q\cdot r\right)\rho_{c}^{\left(\lambda \right)}\left(r\right)dr.$$

**Figure 1:**
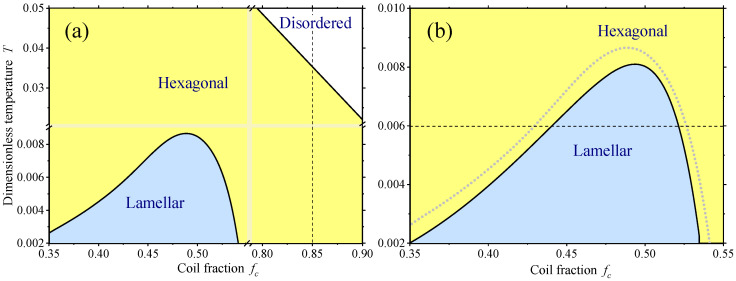
Coil fraction $f_{c}$ – dimensionless temperature $T={\left(\beta J_{0}\right)}^{-1}$ phase diagrams of the rod-coil diblock copolymer calculated numerically for N=10, χ=2, and $J_{2}=J_{0}/3$: the diagram obtained by numerical minimization of the free energy with respect to all order parameters (**a**); and a part of the diagram obtained for low $f_{c}$ within the S=1 approximation as discussed in the text. The dashed straight lines correspond to the order parameter profiles presented in Figure 2. The dotted curve in (**b**) represents the lamellar-hexagonal boundary calculated as in (**a**) without using the approximation of perfect orientational order.

Orientational and translational order parameters of rod and coil monomers in different phases have been calculated by direct minimization of the free energies of lamellar (31) and hexagonal (45) phases using the method of Lagrange multipliers. A representative phase diagram containing the isotropic, the hexagonal and the lamellar phases is presented in Figure 1a. One notes that at relatively high coil fraction the hexagonal phase is predominant as the rods have a strong tendency to separate into cylinders. In contrast, at relatively low coil fraction and low temperatures the lamellar phase is generally more stable than the hexagonal one. It is also interesting to note that the system undergoes a reentrant transition from the lamellar into the hexagonal phase with the decreasing coil fraction. Finally, the rod-coil diblock copolymer undergoes a transition into the disordered phase as sufficiently high coil fraction and high temperatures. One notes that the concentration-temperature phase diagram presented in Figure 1 is naturally different from the concentration—Flory-Huggins parameter phase diagrams, presented, for example, in [23,24,35]. The free energies used there are athermal, while in the present theory the contribution of the orientational interaction between the rods depends on temperature as it is determined by intermolecular attraction. At the same time, the Flory-Huggins parameter is assumed to be constant. This concentration-temperature phase diagram is closer to typical diagrams of liquid crystal mixtures which undergo a sequence of transitions into different ordered phases with the decreasing temperature starting from the isotropic phase.

**Figure 2:**
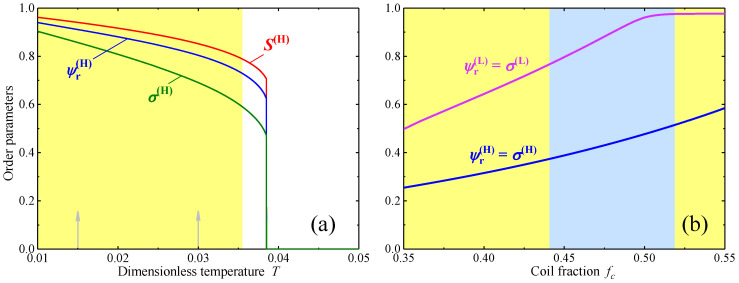
Variations of the orientational and translational order parameters across the phase diagrams shown in Figure 1: order parameters of the hexagonal phase as functions of the dimensionless temperature for $f_{c}=0.85$ (along the vertical dashed line in Figure 1a) (**a**); and the translational order parameters of the lamellar and hexagonal phases as functions of $f_{c}$ resolved within the S=1 approximation for T=0.06 (along the horizontal dashed line in Figure 1b) (**b**). The background colors indicate the regions of stability of the corresponding phases as in Figure 1.

Typical order parameter profiles as functions of temperature in the vicinity of the transition into the disordered phase are presented in Figure 2a for a fixed coil fraction value $f_{c}=0.85$, i.e., for a vertical cross section of the phase diagram shown by the dashed line in Figure 1a. One notes the presence of a narrow range above the transition temperature where the hexagonal ordering corresponds to a local minimum of the free energy being globally unstable with respect to the disordered state. Below the transition temperature, the nematic order parameter *S* rapidly increases reaching the values close to unity. Numerical calculations also reveal that S≈1 in the vicinity of the transition from the hexagonal into the lamellar phase. Thus it is possible to simplify the numerical calculation assuming that S=1 at low coil fractions and low temperatures, i.e., in the region of the phase diagram far from the transition into the disordered phase. The corresponding part of the phase diagram, calculated using the assumption S=1 is presented in Figure 1b. One can readily see that the shape of lamellar phase stability region is very close to the one presented in Figure 1a, where the assumption S=1 has not been employed. For comparison the lamellar-hexagonal boundary calculated without using the approximation S=1 is shown in Figure 1a by the dashed line.

The approximation S=1 has also been used to calculate the profiles of the translational order parameters $\psi_{r}$ as functions of the coil fraction $f_{c}$ at a constant temperature T=0.06 in the lamellar and hexagonal phases presented in Figure 2b. It is interesting to note that the order parameter of the hexagonal phase appears to be significantly smaller than that of the lamellar phase and decreases with the increasing fraction of the rods $f_{r}=1-f_{c}$. This behaviour seems to be counterintuitive but, in fact, it can be explained by packing of rods and coils in the hexagonal structure. Indeed, at low rod fraction, the cylinders composed mainly of rods are relatively thin and the flexible chains, attached to these rods, can escape into the surrounding region with low rod concentration. In this case, the degree of microphase separation is high and the translational order parameter is large. At the same time, at larger rod fraction the effective radius of the cylinders is also larger and, as a result, the coils attached to the rods in the central part of a cylinder, cannot avoid being partially located inside the cylinder as they cannot completely escape into the surrounding region. This inevitable partial mixing of coils with rods inside the cylinders reduces the degree of microphase separation and the values of the translational order parameter $\psi_{r}^{\left(H\right)}$ shown in Figure 2b.

To characterise the local structure of the periodic inhomogeneous phases we introduce two local parameters: the reduced local rod density

(55) $${\tilde{\rho }}_{r}\left(r\right)=\frac{\int \rho_{r}\left(a,r\right)\;da}{{\;\int \int \;}_{H}\rho_{r}\left(a,r\right)\;drda}\;,$$

where the spatial integration in the denominator is taken over the area H of the hexagonal unit cell, and the local nematic order parameter

(56) $$S\left(r\right)=\frac{\int P_{2}\left(a\cdot n\right)\rho_{r}\left(a,r\right)da}{\int \rho_{r}\left(a,r\right)da}.$$

**Figure 3:**
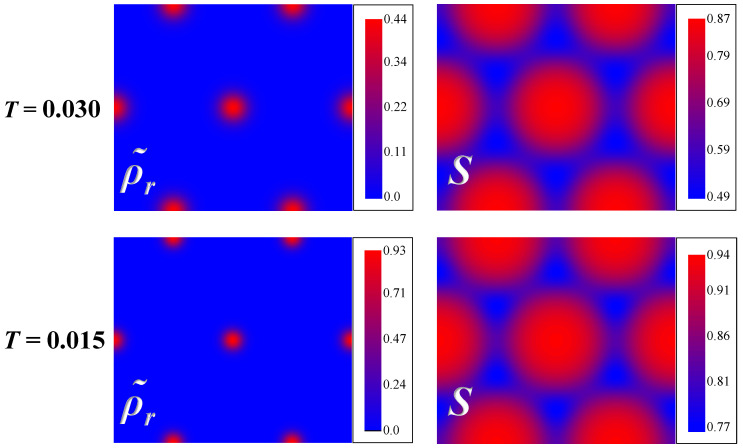
Spatial distributions of the reduced density ${\tilde{\rho }}_{r}$ and the nematic order parameter *S* of the rod-like fragments of copolymer molecules in the hexagonal phase calculated numerically for $f_{c}=0.85$ and for two different temperatures T=0.03 and T=0.015 indicated by the gray arrows in Figure 2a.

Characteristic colourmaps of the spatial distribution of these parameters are shown in Figure 3 for two temperatures indicated by the grey arrows in Figure 2a. One can readily see that the distribution of rods is strongly inhomogeneous and the majority of the rods appear to be densely packed in the central regions of the diffuse cylinders. The local nematic order parameter S(r), however, is rather large also in the regions where the density of rods is small. One may assume that in these regions the orientational order is mainly induced by the structural anisotropy of the hexagonal phase. By evaluating all components of the local nematic tensor order parameter we verify that the director n is parallel to the axis of the cylinders within the accuracy of our numerical procedures. Also the local orientational order of rods remains predominantly uniaxial with the biaxiality of the local tensor nematic order parameter remaining lower than 0.02 across the whole hexagonal structure.

## 6. Discussion and Conclusions

To summarise, it has been shown that the density functional theory of rod-coil diblock copolymers can be used to describe in detail both the microphase separation and the orientational ordering of rod fragments both in the lamellar and in the hexagonal phases. Both orientational and translational order parameters have been calculated numerically by direct minimization of the free energies of various phases The description of the hexagonal phase is a step forward in the density functional theory of rod-coil copolymers as one has to take into consideration the two-dimensional periodicity of the equilibrium densities and the orientation of the director along the six-fold symmetry axis of the phase. The orientational ordering of rod fragments in the hexagonal phase has not been considered before in any detail. In the present theory the orientational ordering of rods is described by two order parameters: the orientational (nematic) order parameter *S* which specifies the degree of the orientational order of rods with respect to the macroscopic director n and the mixed orientational-translational order parameter σ which describes the simultaneous orientational and translational ordering of rod fragments in the form a orientational-density wave. The order parameter σ is widely used in the molecular theory of smectic liquid crystals [50]. The profiles of the order parameters as functions of temperature or fraction of coil monomers $f_{c}$ have been presented for two cross sections of the phase diagrams. It has been shown that the orientational order parameter *S* is very close to saturation both in the hexagonal and in the lamellar phase except for a narrow vicinity of the transition into the disordered phase.

The translational order parameter of the hexagonal phase appears to be much smaller than that of the lamellar phase. Moreover, it decreases with the increasing fraction of the rod monomers $f_{r}$. These properties are related to packing of rods and coils in the hexagonal structure. At low fraction of rod monomers the cylinders, occupied predominantly by rods, are relatively thin and the coils, attached to these rods, can escape into the surrounding space with low density of rods. This results in a high degree of microphase separation and high values of the translational order parameter. In contrast, in the hexagonal phase with large fraction of rod monomers the cylinders are characterised by a larger effective radius and as a result many coils, attached to the rods, located in the central part of a cylinder, cannot escape into the surrounding space and are located partially inside the cylinders. These coils are partially mixed with the rods inside the cylinders which results in the decrease of the microphase separation and the decrease of the translational order parameter.

In the present density functional theory we have used the density-density correlation functions for long Gaussian chains which have been calculated in [24,25]. One notes, however, that many semiflexible polymer chain models, including, in particular, the wormlike chains, cannot be described by the Gaussian chain model because it does not account for the effects of finite rigidity. In principle, the density functional approach can also be applied to these more general models provided some expressions for the correlation functions can be obtained analytically or numerically. The pair density-density correlation functions employed in the present theory also determine the structure factor of the chain. For a wormlike polymer chain the structure factor has been considered by a number of authors [52,53,54,55,56,57] although no reliable analytical expression has been obtained so far. Recently an efficient numerical approach to calculate the structure factor of a wormlike chain has been proposed by Zhang et al. [57] using a formal solution for the Green’s function valid for any chain rigidity. The paper [57] also contains a review of the previous approaches. The results of Monte-Carlo simulations of the discretized wormlike chains which can be used to obtain the structure factor numerically have also been presented recently [58,59,60]. Some of these approaches can, in principle, be used also to evaluate the cross correlation functions between different semi-flexible chains within a single copolymer macromolecule which are required by the present theory in order to describe also the phase separation and orientational ordering in the system of worm like chains.

One notes that the orthogonal lamellar phase is the direct analogue of the liquid crystal smectic A phase which has exactly the same symmetry. At the same time, the hexagonal phase is unique for block copolymers (both coil-coil and rod-coil) and is not exhibited by conventional thermotropic liquid crystals. There exist the smectic B and smectic H liquid crystal phases which possess the hexagonal symmetry but these phases are characterised by the one-dimensional periodicity with the hexagonal lattice or bond orientational order in each smectic layer. There exists also the so called columnar liquid crystal phase where disc-like mesogenic molecules are self assembled into columns which form a two-dimensional hexagonal lattice. All of these liquid crystal phases are very much different from the hexagonal phase, exhibited by block copolymers, where the hexagonal structure is stabilised by the local separation between rod and coil monomers. The results of the density functional theory indicate that the hexagonal phase is stabilised at low temperatures and at relatively low fraction of rod monomers, and the direct transition from the isotropic into the hexagonal phase is possible.

## Abbreviations

The following abbreviations are used in this manuscript:

SCFT	self-consistent field theory

## Appendix A. Correlation Functions of Noninteracting Rod-Coil Copolymer Chains

The coil-coil density-density correlation function for the reference disordered phase of block copolymers has been calculated by several authors [25,31,32] for long Gaussian chains:

(A1) $$G_{cc}\left(q\right)=\rho_{0}\frac{1}{N}\int_{0}^{Nf_{c}}\int_{0}^{s}\exp\left[\left(s-s^{\prime }\right)q^{2}a^{2}/6\right]dsds^{\prime }=\rho_{0}N\frac{2}{x^{2}}\left[f_{c}x+\exp\left(-f_{c}x\right)-1\right],$$

where $f_{c}$ is the fraction of coil segments, $x=q^{2}Na^{2}/6=q^{2}R^{2}$ and it has been assumed that $f_{c}N\gg 1$.

The rod-coil and rod-rod density correlation functions for diblock copolymers, averaged over all orientations of the rod segment, have been calculated in [24]. The corresponding unaveraged correlation functions $G_{rc}\left(q,a\right)$ and $G_{rr}\left(q,a\right)$, which depend on the orientation of the rod segment of a diblock molecule, can be calculated using general expressions derived in [24]. In particular, the rod-coil correlation function can be expressed as:

(A2) $$G_{rc}\left(q,a\right)=\rho_{0}Nf_{r}f_{c}K_{R}^{\left(1\right)}\left(y\right)K_{c}^{\left(1\right)}\left(x\right),$$

where

(A3) $$K_{c}^{\left(1\right)}\left(x\right)=\frac{1}{x}\left[1-\exp(-x)\right],$$

and

(A4) $$K_{R}^{\left(1\right)}\left(y\right)=Re\frac{1}{Nf_{r}}\int_{0}^{Nf_{r}}\exp\left[i\left(q\cdot a\right)s\right]ds=\frac{\sin(y)}{y},$$

where $y=Nf_{r}qa\left(k\cdot a\right)$ and where $f_{r}$ is the fraction of rod segments.

The rod-rod correlation function can be written in the form:

(A5) $$G_{rr}\left(q,a\right)=\rho_{0}\frac{1}{N}\int_{0}^{Nf_{r}}\int_{0}^{Nf_{r}}\exp\left[i\left(s-s^{\prime }\right)qa\left(q\cdot a\right)\right]dsds^{\prime }=2\rho_{0}Nf_{r}^{2}\frac{\left(1-\cos y\right)}{y^{2}}.$$

The direct correlation functions $C_{\nu \mu }$ of the disordered reference phase of non-interacting chains are given by the general Equations (22)–(24) derived above. In particular, it yields the following explicit expressions:

(A6) $$C_{rr}^{\left(0\right)}\left(q,a\right)=\frac{1}{\rho_{0}f_{r}}-\frac{G_{cc}^{\left(0\right)}}{Det^{\left(0\right)}}$$

(A7) $$C_{cc}^{\left(0\right)}\left(q,a\right)=\frac{1}{\rho_{0}f_{c}}-\frac{G_{rr}^{\left(0\right)}}{Det^{\left(0\right)}}$$

(A8) $$C_{rc}^{\left(0\right)}\left(q,a\right)=\frac{G_{rc}^{\left(0\right)}}{Det^{\left(0\right)}},$$

where

(A9) $$Det^{\left(0\right)}=G_{rr}^{\left(0\right)}G_{cc}^{\left(0\right)}-{\left(G^{\left(0\right)}\right)}_{rc}^{2}.$$

where

(A10) $$G_{rr}^{\left(0\right)}\left(q\right)=\int G_{rr}\left(q,a\right)da,$$

(A11) $$G_{cc}^{\left(0\right)}\left(q\right)=\int G_{cc}\left(q,a\right)da,$$

(A12) $$G_{rc}^{\left(0\right)}\left(q\right)=\int G_{rc}\left(q,a\right)da.$$

Finally the functions $C_{\nu \eta }^{\left(2\right)}\left(q\right)$ can be written in the form:

(A13) $$\begin{array}{c} C_{rr}^{\left(2\right)}\left(q\right)=C_{rr}^{\left(1\right)}\left(q\right)-C_{rr}^{\left(0\right)}\left(q\right), \end{array}$$

(A14) $$\begin{array}{c} C_{cc}^{\left(2\right)}\left(q\right)=C_{cc}^{\left(1\right)}\left(q\right)-C_{cc}^{\left(0\right)}\left(q\right), \end{array}$$

(A15) $$\begin{array}{c} C_{rc}^{\left(2\right)}\left(q\right)=C_{rc}^{\left(1\right)}\left(q\right)-C_{rc}^{\left(0\right)}\left(q\right), \end{array}$$

where the functions $C_{\nu \eta }^{\left(1\right)}\left(q\right)$ are obtained by setting (a·k)=1 in the equations for the functions $C_{\nu \eta }\left(q\right)$ and hence

(A16) $$C_{rr}^{\left(1\right)}\left(q\right)=\frac{1}{\rho_{0}f_{r}}-\frac{G_{cc}^{\left(1\right)}}{Det^{\left(1\right)}}$$

(A17) $$C_{cc}^{\left(1\right)}\left(q\right)=\frac{1}{\rho_{0}f_{c}}-\frac{G_{rr}^{1}}{Det^{\left(1\right)}}$$

(A18) $$C_{rc}^{\left(1\right)}\left(q\right)=\frac{G_{rc}^{\left(1\right)}}{Det^{\left(1\right)}},$$

where

(A19) $$Det^{\left(1\right)}=G_{rr}^{\left(1\right)}G_{cc}^{\left(1\right)}-{\left(G_{rc}^{\left(1\right)}\right)}^{2}.$$

Here the functions $G_{rr}^{\left(1\right)},G_{cc}^{\left(1\right)},G_{rc}^{\left(1\right)}$ are given by Equations (A1)–(A5) with $y=y^{*}=Nf_{r}q^{*}$.
